# Ameliorative Effect of Thymoquinone-Loaded PLGA Nanoparticles on Chronic Lung Injury Induced by Repetitive Intratracheal Instillation of Lipopolysaccharide in Rats

**DOI:** 10.1155/2021/5511523

**Published:** 2021-05-28

**Authors:** Sultan A. M. Saghir, Naif A. Al-Gabri, Abdelmoniem A. Ali, Al-Sayed R. Al-Attar, Mosa'd Al-Sobarry, Omar Y. A. Al-shargi, Amal Alotaibi, Mohammed Al-zharani, Fahd A. Nasr, Nader Al-Balagi, Mahfoudh A. M. Abdulghani, Sulaiman M. Alnaimat, Osama Y. Althunibat, Ayman M. Mahmoud

**Affiliations:** ^1^Department of Medical Analysis, Princess Aisha Bint Al-Hussein College of Nursing and Medical Sciences, Al-Hussein Bin Talal University, Ma'an 71111, Jordan; ^2^Department of Pathology, Faculty of Veterinary Medicine, Thamar University, Dhamar 87246, Yemen; ^3^Department of Pathology, Faculty of Veterinary Medicine, Zagazig University, Zagazig, Egypt; ^4^Laboratory of Djibouti Regional livestock Quarantine, Abu Yasser International Est., Djibouti; ^5^Department of Pharmacology, College of Pharmacy, Ittihad Private University, Al-Raqqah, Syria; ^6^College of Pharmacy, Riyadh Elm University, Riyadh, Saudi Arabia; ^7^Basic Science Department, College of Medicine, Princess Nourah bint Abdulrahman University, Riyadh 11671, Saudi Arabia; ^8^Biology Department, College of Science, Imam Mohammad ibn Saud Islamic University, Riyadh 11623, Saudi Arabia; ^9^Medicinal, Aromatic and Poisonous Plants Research Center, College of Pharmacy, King Saud University, Riyadh 11451, Saudi Arabia; ^10^Ministry of Health, Riyadh, Saudi Arabia; ^11^Department of Pharmacology & Toxicology, Unaizah College of Pharmacy, Qassim University, Al Qassim 51911, Saudi Arabia; ^12^Biotechnology Department, Research Institute of Medicinal and Aromatic Plants, Beni-Suef University, Beni-Suef, Egypt; ^13^Physiology Division, Zoology Department, Faculty of Science, Beni-Suef University, Beni-Suef, Egypt

## Abstract

Thymoquinone (TQ), the active constituent of *Nigella sativa*, possesses several benefits in traditional and modern medicines. This study examined the effect of a single dose of Nano-TQ on chronic lung injury induced by repetitive intratracheal installation of lipopolysaccharide (LPS). Rats received LPS twice weekly for 8 weeks via intratracheal installation and a single dose of TQ-PLGA NPs on the day after the last dose of LPS. Six rats from each group were sacrificed after 8 and 10 weeks, and samples were collected for analysis. Repetitive intratracheal installation of LPS caused histopathological alterations, including partial or complete obstruction of the alveoli, interstitial edema, mild fibroblastic proliferation, fibrous strands besides lymphocytes and plasma infiltrations, suffered fetalization, bronchiectasis, hypertrophied arterioles, and others. Investigation of the ultrastructure revealed prominent necrotic pneumocytes with destructed chromatin and remnant of necrotic debris in the narrowing alveolar lumen in LPS-induced rats. TQ-PLGA NPs effectively ameliorated LPS-induced histopathological and ultrastructural alterations in the lung of rats. In addition, TQ-PLGA NPs significantly alleviated serum levels of IL-10 and TGF-*β*1 in LPS-induced rats. In conclusion, TQ-PLGA NPs prevented inflammation and tissue injury in the lungs of rats challenged with repetitive intratracheal installation of LPS. Therefore, TQ-PLGA NPs represent a promising candidate for the prevention of lung injury induced by LPS, pending further studies to determine its safety and exact protective mechanism.

## 1. Introduction

Chronic airway inflammation leads to respiratory diseases such as chronic obstructive pulmonary disease (COPD), resulting in a significant economic and social burden [1, 2]. Chronic airway inflammation provokes persistent lung injury (PLI) caused by bacterial infection [3, 4]. A large number of virulent microbes requires the activation of innate immune system, mainly neutrophils and macrophages. In PLI, multiple inflammatory cells and mediators are activated, and the mechanisms for resolving inflammation are impaired [1, 5–7]. In response to microbial infection, Toll-like receptors (TLRs) mediate the activation of macrophages. TLRs are pattern recognition receptors (PRRs) and fundamental regulators of both innate and adaptive immunity [8, 9]. Lipopolysaccharide (LPS), the main component of the bacterial cell wall [10, 11], stimulates TLR-4 and promotes the activation of nuclear factor *κ*B (NF-*κ*B) and subsequent release of proinflammatory cytokines [12]. Thus, suppressing the NF-*κ*B signaling pathway can represent a strategy to downregulate excessive inflammatory responses. In pulmonary disease, cytokines and inflammatory factors, such as transforming growth factor-beta (TGF-*β*1) and interleukin- (IL-) 10, are released from the injured tissues and promote lung fibrosis [13, 14]. TGF-*β*1 is considered the key factor behind pulmonary fibrosis [15]. Besides its effect in the transdifferentiation of quiescent fibroblasts into myofibroblasts, it is the basic inducer of alveolar epithelial–mesenchymal transformation [16]. Interestingly, the activation of TGF-*β*1 is regulated by IL-10, which can play a part in attenuating the fibrosis [17].

Thymoquinone (TQ) is the primary active chemical component of the *Nigella sativa* essential oil. TQ is a potent antioxidant and anti-inflammatory agent [18–20] and a potential therapeutic effect in respiratory diseases [21–23]. TQ protects against toxic medications such as bleomycin-induced lung fibrosis [24, 25], gentamicin-induced kidney and liver injury [19, 20], and titanium dioxide nanoparticle-induced toxicity [26]. However, it has limitations in effectiveness due to its narrow therapeutic window and poor oral bioavailability [27, 28]. TQ is a hydrophobic molecule and has poor water solubility and poor formulation. TQ-nanoparticles (TQ NPs) revealed potential antioxidant and anti-inflammatory effects [29, 30] and improved oral bioavailability and formulation [21, 31]. Poly lactic-co-glycolic acid (PLGA) improved the pharmacokinetics and pharmacodynamics properties of TQ NP intranasal administration [32]. In addition, a prior study reported that TQ-PLGA NPs possess the capability to attenuate bleomycin-induced pulmonary fibrosis through suppression of oxidative stress and inflammation [33]. Accordingly, this study was established to explore the ameliorative effects of TQ-PLGA-PVA NPs on chronic lung injury induced by repetitive intratracheal (i.t.) instillation of LPS in rats and its effects on regulation of serum TGF-*β*1 and IL-10 levels.

## 2. Materials and Methods

### 2.1. Designing TQ-PLGA Nanoemulsion

**Figure 1:**
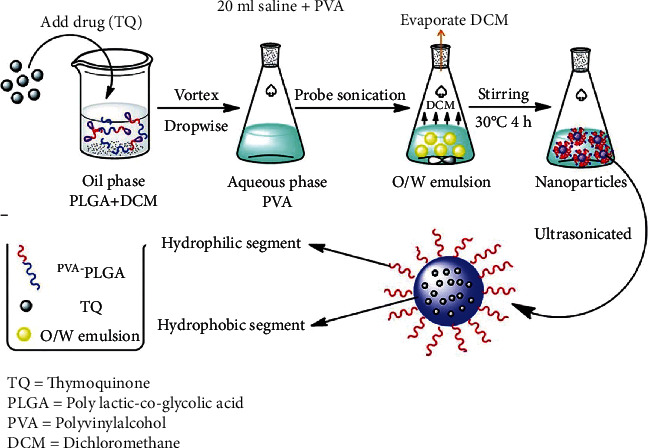
A schematic illustration of TQ-PLGA NP synthesis method.

Nanoparticles were prepared with PLGA using the solid/oil/water (S/O/W) solvent evaporation method as previously described [21, 34] with some modifications (Figure 1). Briefly, PLGA (Sigma, USA) was dissolved in HPLC-grade dichloromethane (DCM) as an oil phase for 12 h to obtain uniform solution followed by the addition 5 mg TQ (Sigma, USA). The suspension was sonicated for 2 min to generate S/O primary emulsion that was emulsified with an aqueous phase of 20 ml saline with polyvinyl alcohol (1% *w*/*v*) to form S/O/W emulsion using a magnetic stirrer at 400 rpm. The mixture was vortexed for 10 sec at a high setting followed by ultrasonication (20 KH2) for 3 min to generate the final S/O/W emulsion. The organic solvent was evaporated using a rotary evaporator at 50°C. Following centrifugation at 10,000 rpm for 20 min at 4°C, the synthesized material was collected and resuspended in 2% sucrose. The shape and size of the particles were confirmed using transmission electron microscope (TEM) [35]. The characteristics of TQ-PLGA NPs were reported in our previous work [33]. The suspension of TQ-PLGA NPs exhibited turbid white color, and the nanoparticles were spherical in shape and 20 nm (10-30 nm) sized with 80% encapsulation efficiency.

### 2.2. Encapsulation Efficiency (EE) of TQ-PLGA NPs

Nanoparticles suspension was subjected to centrifugation at 30,000 rpm for 15 min. The supernatant was removed, and 1 ml of methanol was added to the sediment which was treated with sonication for 5 min and then injected to HPLC to measure the amount of TQ. The EE was 80% as calculated using the following equation [21]:

(1) $$\begin{array}{c} \text{EE}\%=\frac{\text{Total TQ}-\text{Nonencapsulated TQ}}{\text{Total TQ}}\times 100 \end{array}$$

### 2.3. Animals and Treatments

Forty-eight male healthy albino rats weighing 250 ± 50 g, obtained from the animal house of Zagazig University, were used in this investigation. The animals were housed under standard conditions and given a free access to water and a standard diet (El-Nasr Chemical Company, Egypt). The study was conducted according to the guidelines of the National Institutes of Health (NIH publication No. 85-23, revised 2011) and were approved by the local animal care review committee of Zagazig University (Ethical approval number: ZU-IACUC/2/F/71/2020).

The animals were acclimatized for one week and allocated into 4 groups as follows: Group I (control): received i.t. instillation of saline twice weekly for 8 weeks. Group II (TQ-PLGA NPs): received i.t. instillation of saline twice weekly for 8 weeks and a single i.t. dose of TQ-PLGA NPs on the day after the final dose of saline. Group III (LPS): received i.t. instillation of LPS dissolved in saline twice weekly for 8 weeks. Group IV (LPS + TQ-PLGA NPs): received i.t. instillation of LPS dissolved in saline twice weekly for 8 weeks and a single i.t. dose of TQ-PLGA NPs on the day after the final dose of LPS.

LPS and TQ-PLGA NPs were i.t. instilled as previously described [36, 37] with slight modifications. In brief, ketamine/xylazine-anesthetized rats were fixed on their back at an angle of 70 degrees on a glass board. LPS (*Escherichia coli* 055: B5; Sigma, USA) and TQ-PLGA NPs were dissolved in saline and instilled into the trachea at a dose level of 2 mg/kg body weight [33]. i.t. instillation was performed using a 3-gauge intravenous plastic needle connected to a syringe followed by 0.3 ml air. The rats were then placed in a vertical position and rotated for 1 min to distribute the instillation evenly within the lungs.

Six rats from each group were sacrificed under ketamine/xylazine anesthesia 24 h after the instillation of TQ-PLGA NPs (8 weeks point) and at the end of week 10 (10 weeks point) from the beginning of the experiment. The gross lesions were recorded, and blood samples were collected via cardiac puncture, left to coagulate for 30 min, and centrifuged at 3000 rpm for 10 min, and serum was separated. Specimens from lungs were fixed in 10% neutral buffered formalin for histopathology, and other specimens were rapidly kept in 2.5% glutaraldehyde for TEM.

### 2.4. Histopathology

The examined tissue specimens were fixed in 10% buffered formalin for 24 h. Preserved tissues were processed routinely by the paraffin embedding technique [38]. Histopathological changes were evaluated by light microscopy by semiquantitative lesion score system. Lesion score represented by -, normal; +, mild; ++, moderate; and +++, severe as previously described [39].

### 2.5. Ultrastructure Assessment

To examine the ultrastructure, the lung samples were rapidly trimmed into fine sections by sharp blade and fixed in 2.5% glutaraldehyde (pH 7.2) for 4 h then put in 1.33% osmium tetroxide overnight at 4°C followed by dehydration and clearance. The samples were embedded in epoxy resin, and sem-thin sections were obtained by ultramicrotome and stained by toluidine blue and evaluated by light microscopy to detect lesions [40]. The sections were processed for the detection of lesions after the tissue up-load on the grid and stained by lead citrate and uranium. Finally, the ultrasections were evaluated by TEM (JEOL JEM-1230).

### 2.6. Assessment of TGF-*β*1 and IL-10

Sera levels of IL-10 were assayed using a specific ELISA kit (Cat. No. ELR-IL-10, RayBiotech, USA), and TGF-*β*1 was measured by the ELISA kit (Cat. No. K4344, BioVision, USA) according to the manufacturers' instructions. The sensitivity of TGF-*β*1 ELISA kit is <1 pg/ml, whereas the sensitivity of IL-10 kit is 10 pg/ml.

### 2.7. Statistical Analysis

The results of IL-10 and TGF-*β*1 were statistically analyzed using two-way ANOVA on GraphPad Prism 7 followed by Tukey's test. The results are presented as means ± standard error of the mean (SEM), and *P* < 0.05 was considered statistically significant.

## 3. Results

### 3.1. Clinical Signs and Gross Findings

**Figure 2:**
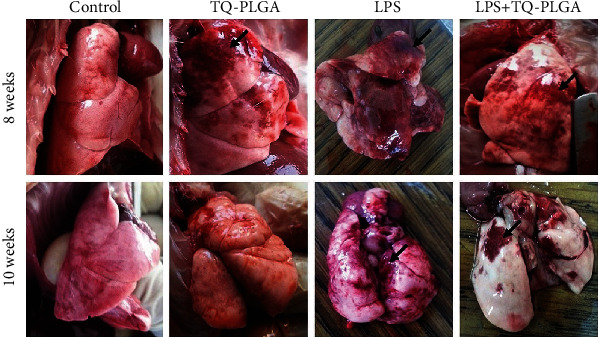
The gross pictures from control rats showing normal morphology of pulmonary tissues, TQ-PLGA NP group showing mild apical hepatized area (arrow), LPS-challenged rats showing consolidations as a depressed area and in small-size lobules (arrows), and LPS + TQ-PLGA NPs showing restoration of the normal size of the lobules with still small red foci (arrows).

The rats did not suffer any clinical signs or mortality throughout the experiment. The control group showed normal morphology of pulmonary tissues at 8 and 10 weeks. Supplementation of TQ-PLGA NPs to normal rats exerted no alterations, and only the focal mild apical hepatized area was observed. The gross pictures showed the effect of repetitive i.t. instillation of LPS at 8 and 10 weeks that revealed consolidations as a depressed area and in general small-size lobules. Moreover, TQ-PLGA NPs restored the normal size of lobules with still small red foci in the lung of LPS-challenged rats (Figure 2).

### 3.2. Histopathological Findings

**Figure 3:**
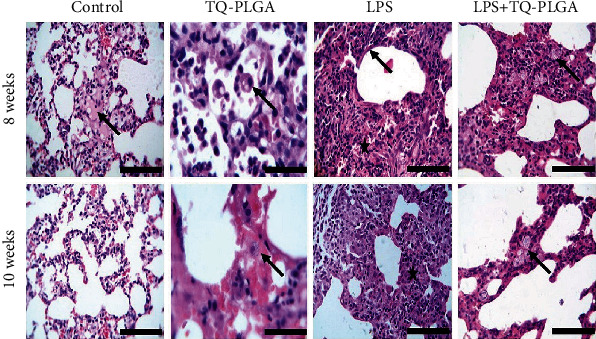
Representative photomicrograph of the control rats showing normal histomorphology architectures with mild edema and congested blood vessels at 8 weeks (arrow) and normal pulmonary alveoli and septa with minute congested blood vessels at 10 weeks, TQ-PLGA NP group showing activated alveolar macrophages (arrow) besides little acute inflammatory cells in the alveolar lumen at 8 weeks and hemorrhages with congested blood capillary (arrow), LPS-challenged rats showing marked obstructions of pulmonary alveoli due to fibroblastic proliferations and chronic inflammatory cells (star) with hyaline wall (arrow) at 8 weeks, and alveolar obstruction with moderate thickening septa (star) at 10 weeks, and LPS + TQ-PLGA NPs showing severe thickening of alveolar septa characterized by proliferated pneumocytes admixed with chronic inflammatory cells which contain granular nanoparticles (arrow) at 8 weeks, remodeling the pulmonary tissues characterized with clear lumen and nearly thinning alveolar septa which contain nanomaterials (arrow) (H&E, scale bar 100 *μ*m).

**Figure 4:**
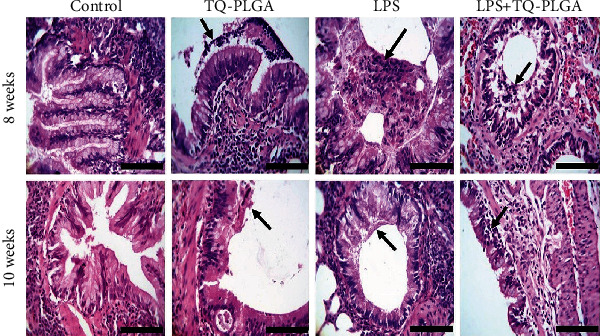
Representative photomicrograph of the pulmonary airways of the control rats showing normal columnar epithelium with mild goblet cell metaplasia and normal pulmonary airways, TQ-PLGA NP group showing clear mucous inflammatory exudate intact to the bronchial mucosa (arrow) at 8 weeks and submucosal vacuoles with few desquamated cells (arrow) at 10 weeks, LPS-challenged rats showing partial obstructions of the bronchioles due to thick mucus materials admixed with inflammatory cells (arrow) at 8 weeks and narrow airways due to narrow airways due to mucous secretions pavementing on the surface of normal epithelium lining (arrow) at 10 weeks, and LPS + TQ-PLGA NPs showing little mucus with inflammatory cells (arrow) and still peribronchial hemorrhages at 8 weeks and nearly normal pulmonary airways with clear lumen and still slight submucosal edema and a few lymphocytes (arrow) (H&E, scale bar 100 *μ*m).

**Figure 5:**
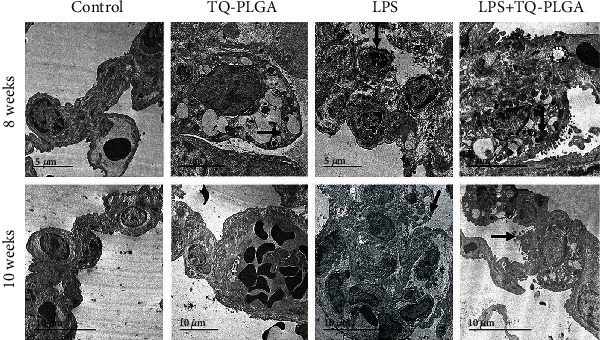
Representative electron micrograph of the lung of control rats showing normal ultrastructure (arrow) at 8 weeks and 10 weeks, TQ-PLGA NP group showing nearly normal pneumocytes nuclei with prominent vacuolated cytoplasm with a little lamellar material as well as nanomaterials (arrow) at 8 weeks and normal air-blood barrier with widening of blood capillary lumen which contain numerous erythrocytes (arrow) at 10 weeks, LPS-challenged rats showing diffuse loss of rough endoplasmic reticulum, mitochondria, blebbing of the nuclear membrane and glycogen lysis with destructed chromatin (arrows) at 8 and 10 weeks, and LPS + TQ-PLGA NPs showing some collagen fibers deposits besides vacuolated pneumocytes which contain nanomaterials (arrow) at 8 weeks, and prominent restoration of the ultrastructural features of the pneumocytes ultrastructure (arrow) at 10 weeks.

Examined sections from sacrificed rats received repetitive i.t. saline revealed normal histomorphology architectures of both alveoli and airways with mild edema and congestions at week 8. After 10 weeks, all structures were normal without any alterations except slight individual congested blood vessels and limitation emphysema (Figures 3–5). Sections from sacrificed rats treated with TQ-PLGA NPs at 8 weeks revealed activated alveolar macrophages which contain NPs and nearly normal airways except a few exfoliated little mucosa inside the lumen. Moreover, nearly normal blood vessels and capillaries with mild vacuolated media followed by edema were observed in few rats. After 10 weeks, the sections revealed congested blood capillary with few pulmonary foamy cells containing NPs, whereas airways contained small vacuoles and blood vessels appeared normal (Figures 3 and 4).

The examined sections from rats received with repetitive i.t. LPS twice weekly for 8 weeks revealed chronic pneumonia represented by chronic exudate within the alveoli and complete or partial obstruction due to interstitial edema and mild fibroblastic proliferation. These alterations along with little fibrous strands beside lymphocytes and plasma infiltrations were observed in the majority of sections. Some alveoli suffered hyperplasia of the alveolar epithelium, and the pulmonary airways showed partial obstructions of the bronchioles (bronchiectasis) due to thick mucus materials admixed with inflammatory cells. Hypertrophied arteriole wall was noticed, and some emphysematous alveoli showed partial hyalinization of their wall. After 10 weeks (two weeks post the last LPS dose), the lesion in the majority of sacrificed rats is still intense and pronounced and represented by diffuse and large chronic active pneumonic areas consisted of necrotic alveolar tissues invaded by proliferated and hyperplastic pneumocytes type II and alveolar macrophages (foamy cells). The airways suffered narrowing due to goblet cells metaplasia (Figures 3 and 4).

Sections from rats treated with TQ-PLGA NPs after LPS and sacrificed 24 h after treatment revealed amelioration of the lung lesions with still thickening of alveolar septa characterized by large foamy cells which contain dark bluish granular nanoparticles besides the absence of exudate in the majority of alveoli lumen. Numerous active macrophages (foamy cells) with vacuolated cytoplasm with tendency to form syncytial giant cells were predominant in interalveolar tissue. The airways still show a little mucous with inflammatory cells on the mucosa and goblet cells metaplasia (Figures 3 and 4).

The histopathological alterations caused by repetitive LPS i.t. instillation and the ameliorative effect of TQ-PLGA NPs are summarized in Table 1.

### 3.3. Ultrastructure Findings

The ultrastructure of the lungs of control and TQ-PLGA NP groups appeared normal with no alterations (Figure 5). Lesion scores of ultrastructure findings of pulmonary airways, blood vessels, alveoli, and pulmonary septa in LPS-treated rats are shown in Table 2. Examination of ultrastructures of lung tissues from rats which received repetitive i.t. LPS revealed prominent necrotic pneumocytes with destructed chromatin and remnant of necrotic debris from the cytoplasmic organelles which extend to the narrow alveolar lumen. After 10 weeks, LPS-challenged rats showed prominent destructed and necrotic pneumocytes without any cytoplasmic organelles with destructed chromatins and microvilli in the pneumocytes surfaces. In contrast, rats that received TQ-PLGA NPs showed few collagen fiber deposits besides vacuolated pneumocytes with empty lamellar bodies which sometimes contain nanomaterials at 8 weeks, and nearly normal pneumocytes nuclei and its chromatin with prominent vacuolated cytoplasm with a little lamellar material as well as nanomaterials as well as prominent restoration of the ultrastructural features of the pneumocytes after 10 weeks (Figure 5).

### 3.4. TQ-PLGA NPs Ameliorate Serum Levels of IL-10 and TGF-*β*1 in LPS-Challenged rats

**Figure 6:**
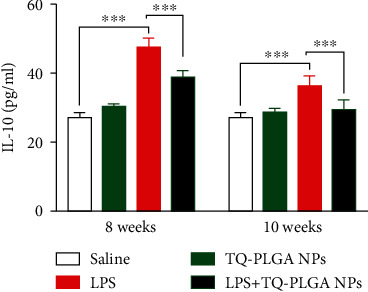
TQ-PLGA NPs ameliorate serum IL-10 in LPS-challenged rats. Data are mean ± SEM, *n* = 6. ^∗∗∗^*P* < 0.001.

**Figure 7:**
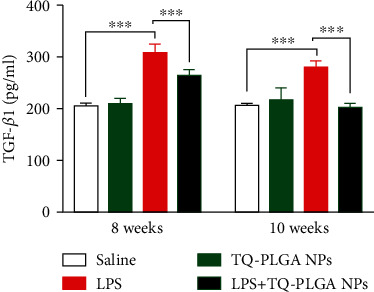
TQ-PLGA NPs ameliorate serum TGF-*β*1 in LPS-challenged rats. Data are mean ± SEM, *n* = 6. ^∗∗∗^*P* < 0.001.

Rats of the LPS group showed a significant increase in serum IL-10 (Figure 6) and TGF-*β*1 (Figure 7) levels when compared with the control rats (*P* < 0.001). Administration of TQ-PLGA NPs ameliorated serum IL-10 and TGF-*β*1 levels in LPS-challenged rats with no effect in normal rats.

## 4. Discussion

Thymoquinone is a promising compound possesses a strong antioxidant and anti-inflammatory activities both *in vitro* and in *vivo*. In the present study, we used a rat model of LPS-induced chronic lung injury to investigate the potential effects of Nano-TQ. Crude TQ could be toxic at high doses and causes allergic dermatitis and has poor water solubility. To overcome these disadvantages, biodegradable and biocompatible polymeric nanoparticles would be attractive alternatives for TQ delivery. The route of administration of Nano-TQ was instillation into the trachea to enhance local TQ effect on pulmonary immune system and avoid systematic injection because it is hydrophobic and should be slowly disseminated [41, 42].

In our experiment, the pulmonary tissue of rats that received repeated doses for LPS was characterized by many chronic alterations, including severe narrowing (bronchostenosis) due to active chronic bronchiolitis with mucous exudates and goblet cell metaplasia. Our results partially agreed with Harkema and Hotchkiss who noticed mucous cell metaplasia in the trachea and bronchial airways of rats exposed to repeated doses of LPS [43]. Similar findings were added by Stolk et al. who described bronchial mucus cell hyperplasia in lungs of hamster due to repetitive i.t. installation of LPS for 5 weeks [44]. Moreover, other researchers noticed thickened airway wall with patchy actin staining after long-term exposure to corn dust extract in mice [45]. On the other hand, our findings included BALT hyperplasia with prominent mitotic and karyorrhexis activities in its germinal center. These findings disagree with Kaneko et al. who attributed thickened airways due to infiltration of leukocytes into BALT [46]. Moreover, the later authors noticed inflammatory cells infiltrations within emphysematous alveoli, swelling of the alveolar walls besides goblet cell hyperplasia in the airways, and a large number of inflammatory cells infiltrations mainly macrophages and neutrophils after 15 min from LPS exposure by nebulizer [46].

The lesions in our work also included emphysema and bronchial mucus cell hyperplasia. Prior study noticed that PMNs recruitment and the neutrophil-derived elastase can induce pulmonary emphysema and bronchial mucus cell hyperplasia [44], findings that are consistent with the current study. Additionally, inhibition of elastase resulted in protection against LPS-induced emphysema and to a lesser extent bronchial mucus cell hyperplasia [44]. Furthermore, Khedoe et al. found emphysema and increased number of macrophages, alveolar destruction, and neutrophils infiltrations in sacrificed mice after LPS administration (twice weekly for 20 weeks) [47]. The findings of this study stated the presence of prominent pulmonary emphysema in rats sacrificed after 8 and 10 weeks which are in agreement with findings of Finlay and his colleges who considered that the presence of emphysema could be due to COPD [48]. They also demonstrated an increase in BALT concentrations and macrophage expression of matrix metalloproteinase-1 (MMP-1) and MMP-9 [48].

Similar conclusions were reported previously indicating that the mechanisms of emphysema include activation of macrophages by LPS release and elastolytic enzymes (proteinase), mainly MMP-2, MMP-9, MMP-12, and cytokines from chemotactic neutrophils [49–51]. Also, emphysema could be responsible for destruction of lung parenchyma, including alveolar wall and capillary beds [51].

Partial or complete obstruction of the alveoli (chronic alveolitis) accompanied with interstitial proliferated and activated pneumocytes together with alveolar macrophages were common in LPS-challenged rats. Similar results were reported by previous investigations showing that chronic alveolitis could be attributed to numerous mononuclear leukocytes infiltrations mainly macrophages in the pulmonary parenchyma and mucus cell metaplasia in airways [52, 53]. Our results pointed out that LPS was a strong activator of macrophages. Moreover, resident macrophages were activated by lymphokines to induce a second burst of cytokines mainly via NF-*κ*B activation [53]. The pulmonary septa had variable degrees of thickness due to fibroblast proliferation besides airways fibrosis. The airway fibrosis was explained by consistent and repetitive LPS administration which led to continuous infiltration and activation of neutrophils which followed by increased expression of TGF-1 in the conducting airways [54].

The current study showed an increase in TGF-*β*1 level in agreement with prior reports showing that cytokines in chronic obstruction diseases are involved in tissue remodeling and increased expression of TGF-*β*1 that activates proliferation of fibroblasts [55, 56]. Also, Savov et al. noticed inflammatory cell infiltration and deposits of fibrin in subepithelial airways and high levels of MMP-9 in the lung tissue post daily exposure of LPS 4 h/day for 8 weeks [57].

The lung of LPS-challenged rats treated with a single dose of TQ-PLGA NPs revealed many ameliorative events and regenerative attempts which remodeled the majority of pulmonary architectures. A few apoptosis of some BALT and lung epithelium was encountered and could be attributed to the antiproliferative effects of TQ via induction of p53-independent apoptosis and through activation of caspase-8, caspase-9, and caspase-3 in the caspase cascade [58]. Moreover, it was reported that TQ could act as an activator for p53 gene and its downstream effecter p21WAFI and ultimately reduced the antiapoptotic protein Bcl-2 [59, 60]. TQ-PLGA NPs' role in the amelioration of chronic lesions, mainly fibrosis, emphysema, and thickened alveolar and pulmonary septa was demonstrated. Our results fully matched with the findings of El-Khouly et al. who reported that TQ alleviated the progression of pulmonary fibrosis induced by bleomycin in rats [61].

TQ counteracted emphysema of alveoli, inflammatory cell infiltration, hyperplastic lymphoid cells in the surrounding bronchioles, and the overexpression of NF-*κ*B in lung tissue in bleomycin-induced rats [61]. The ameliorative benefits of TQ-PLGA NPs recorded in this study were in accordance with Kanter who noticed possible beneficial effects of *N. sativa* seeds on the experimental lung injury of male rats after pulmonary aspirations of different materials [62]. The same study showed that *N. sativa* treatment significantly reduced peribronchial inflammatory cell infiltration, alveolar septal inflammatory cells infiltration, edema, alveolar exudation, interstitial fibrosis, granuloma, and pulmonary necrosis [62].

The ultrastructural changes due to LPS included severe thickening of blood-air barriers, proliferated pneumocytes, fibroblasts, macrophages, and excess collagen fibrils. Pneumocyte type II exhibited apoptotic changes characterized by condensed nuclear chromatin, distorted nuclear membrane, partial loss of cytoplasmic organelles, and shrunken cell membrane. Pneumocyte type II contained cytoplasmic vacuolation with abnormality or empty lamellar bodies, megamitochondria, and partial loss of surface microvilli. Normally, pneumocyte type II covers 5% of the internal surface area of the lung while pneumocyte type I cells cover the remaining surface [63]. Their distribution was defective in chronic lung injury. The findings of this study were in agreement with Chattopadhyay et al. who reported that the surfactant was increased after platelets derived activated factor (strongly stimulator for the surfactant secretion) released by macrophages challenged by LPS [64]. In addition, LPS can also stimulate surfactant secretion by acting directly on pneumocytes type II cells [65].

In this investigation, the activated and proliferated macrophages were prominent due to monocyte differentiation into macrophages inside the alveoli which comes in line with the observation of Domenici-Lombardo et al. who noticed hyperplasia of pneumocytes type II and hypertrophy of interstitial fibroblasts at 48 h post 5 mg/kg LPS instillation in rats [66]. The monocytes appeared inside blood capillaries and interstitium at 12 h then migrated from alveoli and differentiation into macrophages 24 h [66]. The aforementioned results disagreed with Vernooy et al. who reported that macrophages' number was reduced after 1 week recovery from LPS administration (twice weekly for 8 weeks) [52]. However, Ofulue et al. demonstrated that long-term exposure (up to 6 months) to cigarette smoke resulted in increased intra-alveolar macrophages [67]. LPS-challenged rats that received TQ-PLGA NPs revealed normal blood-air barrier, numerous exuded interalveolar macrophages enclosing multiple intracytoplasmic electron-dense bodies (nanoparticles) besides persistence of little collagen fibrils deposits. The majority of collagen deposits suffered from collagenolysis together with a few degenerated pneumocytes type II containing electron-dense bodies and free nanoparticles in the interstitium. The rats sacrificed after 10 weeks showed normal blood-air barrier, normal alveolar pneumocyte I, and pneumocyte type II containing intracytoplasmic vacuoles and condensed mitochondria.

TQ-PLGA NPs used in this study that are characterized by around 20 nm size and spherical shape were observed inside bronchial epithelium, pneumocytes, macrophages, and interstitium. These results were in a partial agreement with previous report by Hara et al. who noticed fluorescein isothiocyanate- (FITC-) conjugated PLGA nanoparticles inside all pneumocytes and endothelium after i.t. installation of rat lung [68]. Moreover, Kapp et al. [69] and Geiser et al. [70] confirmed that ultrafine particles were detected after ultrafine particles are inhaled. Ultrafine particles can cross cellular membranes by nonphagocytic mechanisms of lungs and culture cells and may appear in many compartments of the body, including the liver, heart, and nervous system [70]. In addition, Geiser et al. detected 20 nm titanium dioxide (TiO_2_) nanoparticles in a bronchial associated macrophages phagolysosomes of rats at 24 h post inhalation [71]. They also detected inhaled ultrafine TiO_2_ particles on the luminal side of airways and alveoli, and within capillaries [71]. The rats that received TQ-PLGA NPs only had mild ultrastructure changes represented by vacuolation of mitochondria and appeared denser along with phagocytic vacuoles which contain dense materials in pneumocyte type II, macrophages, and airways epithelium. Our results partially agreed with Geiser who mentioned that the alveolar macrophages have role in the clearance of inhaled micro- and nanoparticles [72]. In addition, similar findings were reported by Penberthy et al. who demonstrated the phagocytic capacity of bronchial epithelial cells in allergic air ways [73].

The ameliorative effect of TQ-PLGA NPs on LPS-induced lung injury was supported by the significant reduction in serum IL-10 and TGF-*β*1. These results are closely linked to the improvement of alveolar macrophages proliferation and activation. In this context, Yanagawa et al. have reported that macrophages secrete IL-10 in human lung cancer patients [74]. An increase in the expression of TGF-*β* which induced proliferation of fibroblasts in lung of rats exposed to LPS for 8 weeks was noted previously [57]. The ameliorative effect of TQ has also been supported by a previous study showing its ameliorative effect on the pulmonary blood vessels damage in LPS-induced acute injury [23]. The effects of TQ were mediated via modulating proinflammatory cytokines [23].

## 5. Conclusions

This study introduces information on the ameliorative effect of TQ-PLGA NPs on chronic lung injury induced by repetitive i.t. instillation of LPS in rats. The administration of a single dose of TQ-PLGA NPs following the 8-week challenge with LPS significantly ameliorated the histological and ultrastructural alterations in the alveoli, airways and pulmonary blood vessels, and decreased serum levels of IL-10 and TGF-*β*1. Given the well-documented beneficial pharmacological activities of TQ, TQ-PLGA NPs can represent a better alternative to overcome the poor solubility and other factors limiting its therapeutic applications.

## Figures and Tables

**Table 1:** Severity of histopathologic lesions recorded in pulmonary airways, blood vessels, and alveoli in LPS-challeneged rats and the ameliorative effect of TQ-PLGA NPs.

		Control	TQ-PLGA	LPS	LPS + TQ-PLGA	Control	TQ-PLGA	LPS	LPS + TQ-PLGA
		8 weeks				10 weeks			
Airways (bronchi and bronchioles)	Submucosal inflammatory cells infiltrations	-	+	+++	++	-	-	+++	++
	Proliferated epithelium and goblet cells metaplasia	-	+	+++	++	-	-	+++	++
	Bronchostenosis	-	-	+++	++	-	-	+++	++
	Peribronchial plasma and fibroblast proliferation	-	+	+++	++	-	-	+++	++
	Hyperplasia and apoptosis of bronchial associated lymphoid tissues (BALT)	-	-	+++	+++	-	-	+++	+
Pulmonary blood vessels	Endotheliosis	-	-	+++	+	-	-	+++	+
	Perivascular edema	-	-	+++	++	-	-	+++	++
	Hyperplasia of smooth muscles	-	-	+++	++	-	-	+++	+
Alveolar tissue (alveoli and blood-air barrier)	Hyperplasia and hypertrophy of pneumocytes I and II	-	+	+++	++	-	+	+++	++
	Activated alveolar macrophage	-	+	+++	++	-	+	+++	++
	Thickening of alveoli septal	-	+	+++	++	-	+	+++	+
	Atelectasis	-	-	+++	++	-	-	+++	+
	Chronic pneumonia	-	-	+++	+	-	-	+++	+
	Compensatory emphysema	-	+	+++	++	-	+	+++	++

No. of examined fields (5 fields/rat). The severity of lesion was graded by estimating the percentage area affected in the entire section. -: absence of lesion, +: 5–25%, ++: 26–50%, and +++: ≥51%.

**Table 2:** Lesion scores of ultrastructure findings of pulmonary airways, blood vessels and alveoli in LPS-challenged rats and the ameliorative effect of TQ-PLGA NPs.

		Control	TQ-PLGA	LPS	LPS + TQ-PLGA	Control	TQ-PLGA	LPS	LPS + TQ-PLGA
		8 weeks				10 weeks			
Airways (bronchi and bronchioles)	Submucosal inflammatory cells infiltrations	-	+	+++	++	-	-	+++	++
	Proliferated epithelium and goblet cells metaplasia	-	+	+++	++	-	-	+++	++
	Bronchostenosis	-	-	+++	++	-	-	+++	++
	Peribronchial plasma and fibroblast proliferation	-	+	+++	++	-	-	+++	++
	Hyperplasia and apoptosis of bronchial associated lymphoid tissues (BALT)	-	-	+++	++	-	-	+++	+
Pulmonary blood vessels	Endotheliosis	-	-	+++	+	-	-	+++	+
	Perivascular edema	-	-	+++	++	-	-	+++	++
	Hyperplasia of smooth muscles	-	-	+++	++	-	-	+++	+
Alveolar tissue (alveoli and blood-air barrier)	Hyperplasia and hypertrophy of pneumocytes I and II	-	+	+++	++	-	+	+++	++
	Activated alveolar macrophage	-	+	+++	++	-	+	+++	++
	Thickening of alveoli septal	-	+	+++	++	-	+	+++	+
	Atelectasis	-	-	+++	++	-	-	+++	+
	Chronic pneumonia	-	-	+++	+	-	-	+++	+
	Compensatory emphysema	-	+	+++	++	-	+	+++	++
Pulmonary septa	Thickening (inflammatory edema)	-	-	+++	++	-	-	+++	+

No. of examined fields (5 fields/rat). The severity of lesion was graded by estimating the percentage area affected in the entire section. -: absence of lesion, +: 5–25%, ++: 26–50%, and +++: ≥50%.

## Data Availability

Data analyzed or generated during this study are included in this manuscript.
